# Diagnostic consistency between admission and discharge of pediatric cases in a tertiary teaching hospital in China

**DOI:** 10.1186/s12887-023-03995-2

**Published:** 2023-04-15

**Authors:** Dangui Zhang, Baoxin Yan, Siqi He, Shuangshuang Tong, Peiling Huang, Qianjun Zhang, Yixun Cao, Zhiheng Ding, William Ba-Thein

**Affiliations:** 1 Research Center of Translational Medicine, Second Affiliated Hospital of Shantou University Medical College, Shantou, P. R. China; 2 Undergraduate Research Training Program (UGRTP), Shantou University Medical College, Shantou, P. R. China; 3 Clinical Research Unit, Shantou University Medical College, Shantou, P. R. China; 4 Department of Microbiology and Immunology, Shantou University Medical College, Shantou, P. R. China; 5 Clinical Research Unit and Dept. of Microbiology and Immunology, Shantou University Medical College, 11/F, Science & Technology Building, 22 Xinling Road, Shantou, 515041 Guangdong P. R. China

**Keywords:** ICD-10, Discrepancy, Discordance, Coding error, Diagnostic error, Pediatric, China

## Abstract

**Background:**

Patient-centered, high-quality health care relies on accurate and timely diagnosis. Diagnosis is a complex, error-prone process. Prevention of errors involves understanding the cause of errors. This study investigated diagnostic discordance between admission and discharge in pediatric cases.

**Methods:**

We retrospectively reviewed the electronic medical records of 5381 pediatric inpatients during 2017–2018 in a tertiary teaching hospital. We analyzed diagnostic consistency by comparing the first 4 digits of admission and discharge ICD-10 codes of the cases and classified them as concordant for “complete and partial match” or discordant for “no match”.

**Results:**

Diagnostic discordance was observed in 49.2% with the highest prevalence in infections of the nervous and respiratory systems (*Ps* < 0.001). Multiple (multivariable) logistic regression analysis predicted a lower risk of diagnostic discordance with older children (aOR, 95%CI: 0.94, 0.93–0.96) and a higher risk with infectious diseases (aOR, 95%CI: 1.49, 1.33–1.66) and admission by resident and attending pediatricians (aOR, 95%CI: 1.41, 1.30–1.54). Discordant cases had a higher rate of antibiotic prescription (OR, 95%CI: 2.09, 1.87–2.33), a longer duration of antibiotic use (*P* = 0.02), a longer length of hospital stay (*P* < 0.001), and higher medical expenses (*P* < 0.001).

**Conclusions:**

This study denotes a considerably high rate of discordance between admission and discharge diagnoses with an associated higher and longer prescription of antibiotics, a longer length of stay, and higher medical expenses among Chinese pediatric inpatient cases. Infectious diseases were identified as high-risk clinical conditions for discordance. Considering potential diagnostic and coding errors, departmental investigation of preventable diagnostic discordance is suggested for quality health care and preventing potential medicolegal consequences.

**Supplementary Information:**

The online version contains supplementary material available at 10.1186/s12887-023-03995-2.

## Background

Diagnosis is a complex multidimensional process involving information gathering via clinical history, physical exams, and diagnostic testing; integration and interpretation of learned information; and hypothesis generation leading to a potential diagnosis, followed by diagnostic modification and refinement and diagnostic verification to reach a final or definitive diagnosis [1]. The whole process requires good clinical and diagnostic reasoning skills.

Patient-centered, high-quality health care relies on the accuracy and timeliness of diagnosis. Failure to establish an accurate and timely diagnosis based on the currently available evidence and thus to timely inform the patient can be considered a diagnostic error [2–4]. Diagnostic errors, either missed, delayed, or wrong diagnoses, have clinical, economic, and medicolegal consequences [1]. Diagnostic errors have a stronger influence on the patient`s outcomes than any other type of medical error, with the impact ranging from no harm to immediate death in case of serious errors [3, 5]. Since diagnostic errors are relatively common in primary care, the National Academy of Medicine (formerly Institute of Medicine) estimated that most adults in the United States will likely experience one diagnostic error in their lifetime [6].

Recognizing the root causes of each diagnostic error is the primary step for intervention. Diagnostic errors are usually identified by expert reviewers through the triggers in electronic medical records (EMR) [5, 7, 8], autopsies in medical disputes [9], or qualitative self-reports [10]. Analysis of consistency between admission and discharge diagnoses is another approach to studying potential diagnostic errors in many studies [8, 11–17]. Those studies mostly focused on adult patients or admission via the emergency departments [8, 11, 12, 18]. Up to 68% of diagnostic discrepancy rate has been reported, with an increased length of hospital stay, ICU admission, readmission rate, morbidity and mortality, and health care expenses as the consequences [8, 14, 17, 19].

Admission and discharge consistency can be investigated using the International Classification of Diseases (ICD) codes [17] or the physician`s written description of diagnoses in EMR [8, 12]. While determining the diagnostic consistency through written descriptions can be labor-intensive, subjective, and requires a good interrater agreement for accuracy, ICD-based assessment is simple, straightforward, and less error-prone, though the accuracy relies on the ICD coders. Thence, the first 3 digits of ICD-9 [17, 18] or the first 4 digits of ICD-10 [16] have been used in previous studies.

Although evidence of diagnostic problems and their consequences in the adult population is abundant, related research and evidence in pediatric settings are scarce [2, 20]. Therefore, this study aimed to describe the diagnostic consistency status of pediatric inpatient cases and the epidemiology of diagnostic discordance, including high-risk clinical conditions, clinical implications, and causes of diagnostic discordance, and the practicability of ICD-10-based diagnostic consistency assessment in a tertiary hospital in mainland China.

## Methods

### Study design and site

This study was a retrospective review of the EMR of pediatric patients admitted during July 2017-June 2018 to the Pediatric Department of a 1500-bed, tertiary teaching hospital affiliated with Shantou University Medical College. The pediatric department was staffed with 46 pediatricians and 122 nurses, providing primary through tertiary care to approximately 550 new inpatients per month with a pediatrician-to-inpatient ratio of 1:12 and seven medical coders in the Medical Records Department, with 6 coders per day on average as of 2018.

### Patients and data

Of 6785 cases identified via the EMR system during the study period, 5381 were included for analysis after exclusion of 1404 cases with missing relevant clinical or ICD-10 information. Patient`s sex and age, physician profile (professional title and clinical responsibility), clinical management (antibiotic prescription), clinical outcomes (complete or partial recovery, hospital transfer, or death), diagnostic information (admission and primary discharge diagnoses in ICD-10 codes), and associated burden (hospital fees and length of hospital stays) were extracted from the hospital EMR.

### Diagnostic consistency assessment and classification (Table 1)

The Chinese version of ICD-10 [21] is the standard encoding method for diagnosis, symptoms, or syndromes in China. In the study hospital, admission and discharge diagnoses in the physician`s narrative discharge summary are reviewed, and ICD codes are assigned, after the physician`s attestation as needed, by a medical coder from the Medical Records Department. Although ICD codes at different levels of precision may be assigned for a particular clinical condition, for example, T23 (burn, wrist and hand), T23.2 (second-degree burn, wrist and hand), T23.212 A (second-degree burn of left thumbnail, initial encounter), as in a previous study [16], we only used first 4 digits of ICD-10 codes (first three digits representing diagnostic category and the 4th digit for disease specification) for comparing diagnoses at admission to the inpatient ward and primary discharge diagnosis. Additionally, we extracted any provisional or differential diagnoses with or without the ICD-10 code at admission for matching with the primary discharge ICD-10 code for identifying errors related to coding or diagnosis.

**Table 1:** Case examples of ICD-10-based diagnostic consistency assessment

Case example	Admission diagnosis (ICD-10)^1^	Primary discharge diagnosis (ICD-10)	Remark^2^	Match between first 4 digits of two ICD codes^3^	
1	Bronchopneumonia (J18.0)	Bronchopneumonia (J18.0)	Incomplete diagnosis, miscoding, or no error?	Complete match	
2	Hand-foot-mouth disease (B08.4)	Hand-foot-mouth disease (B08.4)			
3	Severe anaemia, unspecified (D64.903)	Severe anaemia, unspecified (D64.903)			
4	Carpopedal spasm (R29.000)	Carpopedal spasm (R29.000)			
5	Hyperpyrexia NOS (R50.900)	Hyperpyrexia NOS (R50.900)			
6	Pneumonia, unspecified (J18.900)	Severe viral pneumoniae (J18.802)	No diagnostic error	Partial match	
7	Chronic tic (F95.1)	Tourette syndrome (F95.2)			
8	Thalassaemia, unspecified (D56.900)	Beta thalassaemia (D56.100)			
9	Herpangina (B08.501)	Hand-foot-mouth disease (B08.401)	Diagnostic error		
10	Sepsis (A41.9)	Candidal sepsis (B37.7)	No diagnostic error	No match	
11	Urinary tract infection (N39.0)	Nonorganic enuresis (F98.0)	Diagnostic error		
12	Hand-foot-mouth disease (B08.4)	Enteroviral meningitis (A87.0**†** or G02.0*)	Misspecification		
13	1. Abdominal pain (R10.4) 2. Urinary tract infection	Urinary tract infection (N39.0)	Miscoding		
14	1. Gastritis (K29.1) 2. Upper respiratory tract infection	Bacterial pneumonia (J15.9)	Diagnostic error or upcoding?		
15	1. Sepsis (A41.9) 2. Neck lymphadenitis	Neck lymphadenitis (L04.002)	Resequencing		
16	1. Tetany (R29.000) 2. Febrile seizure 3. CNS infection 4. Purulent tonsillitis	Purulent tonsillitis (J03.901)	Diagnostic error, miscoding, or resequencing?		

^1^ Including secondary and differential diagnoses

^2^ Miscoding (assigning a generic code when information is available for a more specific code), misspecification (misalignment of primary diagnosis with the evidence in the record), resequencing (coding diagnoses reversely), upcoding (assigning codes of higher reimbursement value) (28)

^3^ Complete match (all first 4 digits of ICD-10 code matched), partial match (at least first 3 digits matched), no match (first 3 digits unmatched)

Diagnostic consistency (the state of agreement) was defined herein as “concordant” for complete match (first 4 digits of ICD-10 code matched) plus partial match (at least first 3 digits matched) and “discordant” for no match (first 3 digits unmatched) [17], as shown in the examples in Table 1. In previous studies, “complete/partial match” or “complete match” between admission and discharge diagnoses was considered consistent (or concordant) [8, 12], which can lead to biases in consistency classification. Therefore, to explore the influence of consistency classification on the outcomes related to diagnostic discordance, we conducted a sensitivity analysis by first categorizing the cases using two criteria of concordance—stringent (complete match only) and flexible (complete/partial match) (Supplementary Table 1). As we found no significant differences across the outcome variables between the two criteria, we used the flexible criteria in downstream analyses.

### Data analysis

**Fig. 1:**
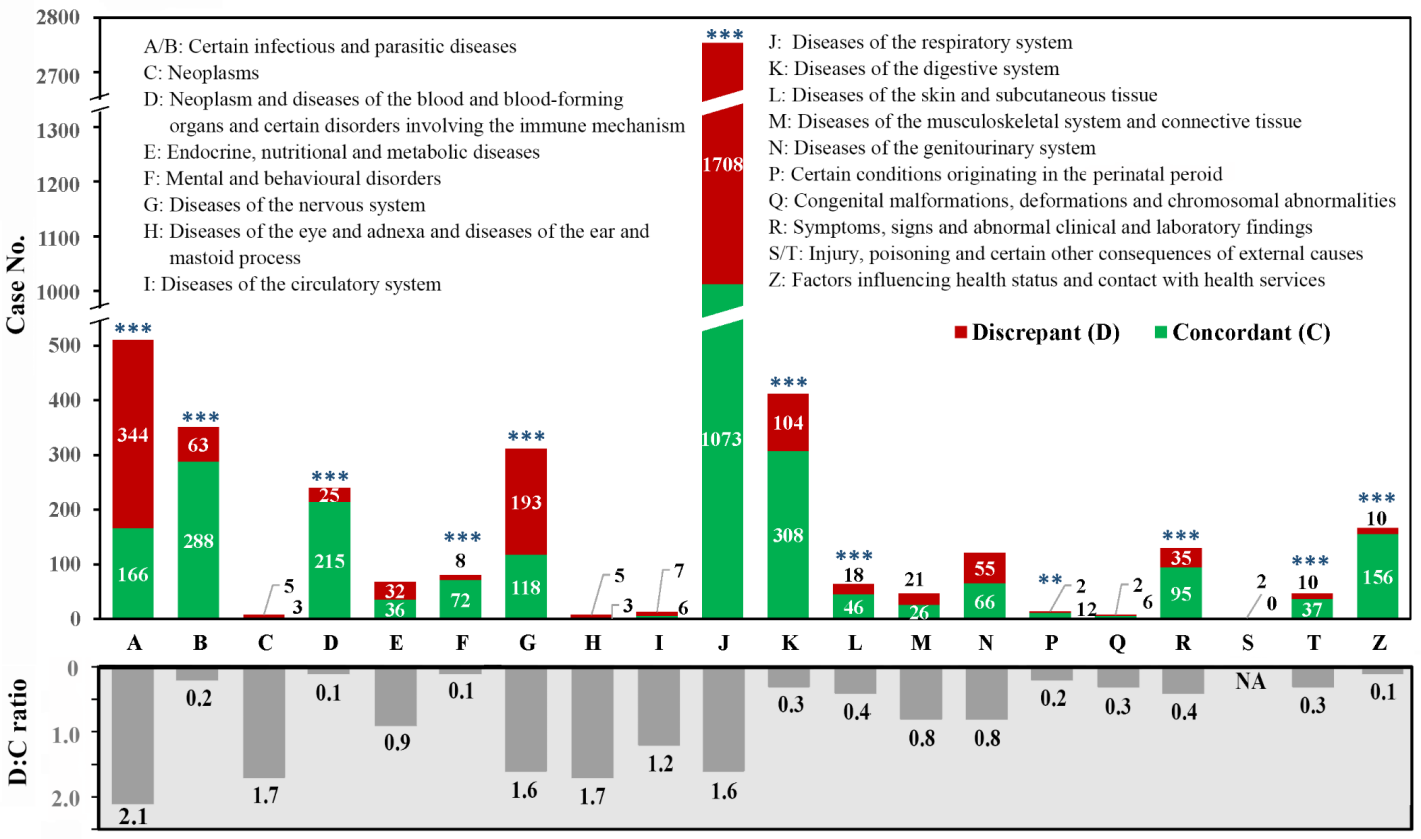
Diagnosis consistency (upper panel) and discrepancy-to-consistency ratio (lower panel) of pediatric cases by ICD-10 group. ** *P* < 0.01, *** *P* < 0.001, consistent vs. discrepant, analyzed by the Chi-square test

Data were extracted from the EMR, classified for diagnostic consistency, and cross-checked for accuracy. Consistency classification was final checked and deliberated by two senior study staff DGZ and WB-T. SPSS v.22 was used for analyzing categorical variables (sex, age group, clinical condition, physician`s title, antibiotic use, outcome) by the Chi-square test and continuous variables (duration of antibiotic use, length of hospital stays, and hospital fees) by the Mann-Whitney U test. The odds of diagnostic discordance in each ICD-10 group are presented as discrepancy-to-consistency ratios (Fig. 1). A multiple (multivariable) logistic regression model was used to analyze the factors associated with diagnosis discordance, which included patients’ age and sex, disease classification such as infectious diseases or others, and the rank and identification of admitting and discharging pediatricians. Two-tailed *P*-values of < 0.05 were considered significant.

## Results

### Characteristics of cases (Table 2)

A total of 5381 admitted cases were studied. The male-to-female ratio was 1.47, with an age range of 28 days to 15 years. Infectious diseases accounted for 45.4% (2443/5381) of the total cases.

**Table 2:** Clinical data of pediatric inpatients and consistency between admission diagnoses and primary discharge diagnoses

Clinical data			Consistency classification		*P* ^3^	OR (95%CI) ^4^
		Total	Concordant ^1^	Discordant ^2^		
		(N = 5381)	n = 2732 (50.8%)	n = 2649 (49.2%)		
**Sex**						
	Male	3207 (59.6)	1634 (59.8)	1573 (59.4)	0.76	1
	Female	2174 (40.4)	1098 (40.2)	1076 (40.6)		1.02 (0.91–1.14)
**Age range** (28 days − 15 years)						
	≥ 3 yr	1853 (34.4)	1047 (38.3)	806 (30.4)	**< 0.001**	1
	2 ~ 3 yr	686 (12.7)	302 (11.1)	384 (14.5)		1.65 (1.38–1.97)
	1 ~ < 2 yr	1063 (19.8)	479 (17.5)	584 (22.0)		1.58 (1.36–1.84)
	< 1 yr	1779 (33.1)	904 (33.1)	875 (33.0)		1.26 (1.10–1.43)
**Admitting pediatrician**						
	Chief	285 (5.3)	225 (8.2)	60 (2.3)	**< 0.001**	1
	Associate chief	927 (17.2)	531 (19.4)	396 (14.9)		2.80 (2.05–3.83)
	Attending	3582 (66.6)	1700 (62.2)	1882 (71.0)		4.15 (3.10–5.56)
	Resident	587 (10.9)	276 (10.1)	311 (11.7)		4.23 (3.05–5.86)
**Discharging pediatrician**						
	Chief	1 (< 0.1)	0 (0)	1 (< 0.1)	0.518	Not relevant
	Associate chief	107 (2.0)	58 (2.1)	49 (1.8)		1
	Attending	2973 (55.2)	1523 (55.7)	1450 (54.7)		1.13 (0.77–1.66)
	Resident	2300 (42.7)	1151 (42.1)	1149 (43.4)		1.18 (0.80–1.74)
**Admitting and discharging pediatricians**						
	Same	225 (4.2)	106 (3.9)	119 (4.5)	0.276	1
	Different	5156 (95.8)	2626 (96.1)	2530 (95.5)		0.86 (0.66–1.12)
**Clinical condition**						
	Non-infectious diseases	2938 (54.6)	1647 (60.3)	1291 (48.7)	**< 0.001**	1
	Infectious diseases	2443 (45.4)	1085 (39.7)	1358 (51.3)		1.60 (1.43–1.78)
**Antibiotic use (Yes)**		2220 (41.3)	890 (32.6)	1330 (50.2)	**< 0.001**	2.09 (1.87–2.33)
**Duration of antibiotic use** (median day, IQR)		5 (4, 7)	5 (4, 7)	5 (4, 7)	**0.02**	
**Length of stay** (median day, IQR)		5 (3, 7)	4 (3, 6)	5 (4, 7)	**< 0.001**	
**Outcome**						
	Recovered completely	3827 (71.1)	1947 (71.3)	1880 (71.0)	0.792	1
	Discharged with partial recovery	1551 (28.8)	783 (28.7)	768 (29.0)		1.02 (0.90–1.14)
	Transferred to another hospital	1 (0.0)	1 (< 0.1)	0 (0)		Not relevant
	Died in hospital	2 (0.0)	1 (< 0.1)	1 (< 0.1)		1.04 (0.07–16.57)
**Hospital fees** (median RMB, IQR)						
	Total	3411 (2282, 5061)	2965 (2011, 4290)	3915 (2652, 5784)	**< 0.001**	
	Drugs only	509 (249, 888)	411 (202, 706)	636 (332, 1040)	**< 0.001**	

Categorical variables (sex, age group, clinical condition, type of pediatricians, antibiotic use, outcome) shown as n (%), analyzed by the Chi-square test; Continuous variables (duration of antibiotic use, length of hospital stay, and hospital fees) shown as median (IQR), analyzed by the Mann-Whitney U test. 1RMB = 0.15 US$; ^1^ Concordant (complete plus partial match); ^2^ Discordance (no match); ^3^ Concordant vs. discordant; ^4^ Risk of discordance

### Diagnostic consistency (Table 2)

Primary discharge diagnoses were completely matched with admission diagnoses in 48.1% (2587/5381) and partially matched in 2.7% (145/5381), whereas no match was observed in 49.2% (2649/5381) of the cases, therefore resulting in 50.8% (2732/5381) of “concordant” and 49.2% of “discordant” diagnostic consistency categories.

Diagnostic consistency significantly differed by age group, admitting pediatricians, and clinical condition (*Ps* < 0.001), with higher odds of diagnostic discordance observable with younger age groups, (OR, 95%CI: 1.26, 1.10–1.43 for < 1 year; 1.58, 1.36–1.84 for 1–2 year; 1.65, 1.38–1.97 for 2–3 year), admission by attending and resident pediatricians (OR, 95%CI: 4.15, 3.10–5.56, and 4.23, 3.05–5.86, respectively), and infectious diseases (OR, 95%CI: 1.6, 1.43–1.78). Diagnostic discordance by the ICD-10 group was most pronounced in the infectious diseases (group A), and the diseases of the nervous system (group G) and respiratory system (group J) with a discordance-to-concordance ratio of 2.1, 1.6, and 1.6, respectively (*Ps* < 0.001, Fig. 1).

**Fig. 2:**
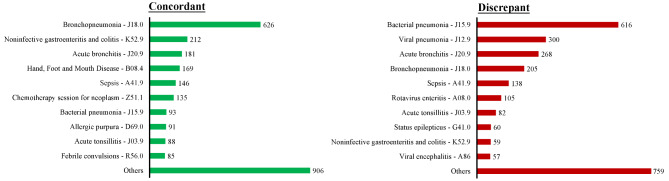
Diagnosis consistency of top 10 primary discharge diagnoses

The top 10 primary discharge diagnoses under concordant and discordant categories were mostly accounted for by infectious diseases (Fig. 2). During the one-year study period, discordance-to-concordance ratios ranged from 0.6 to 1.2, with the highest ratio in January and July (Supplementary Fig. 1).

**Fig. 3:**
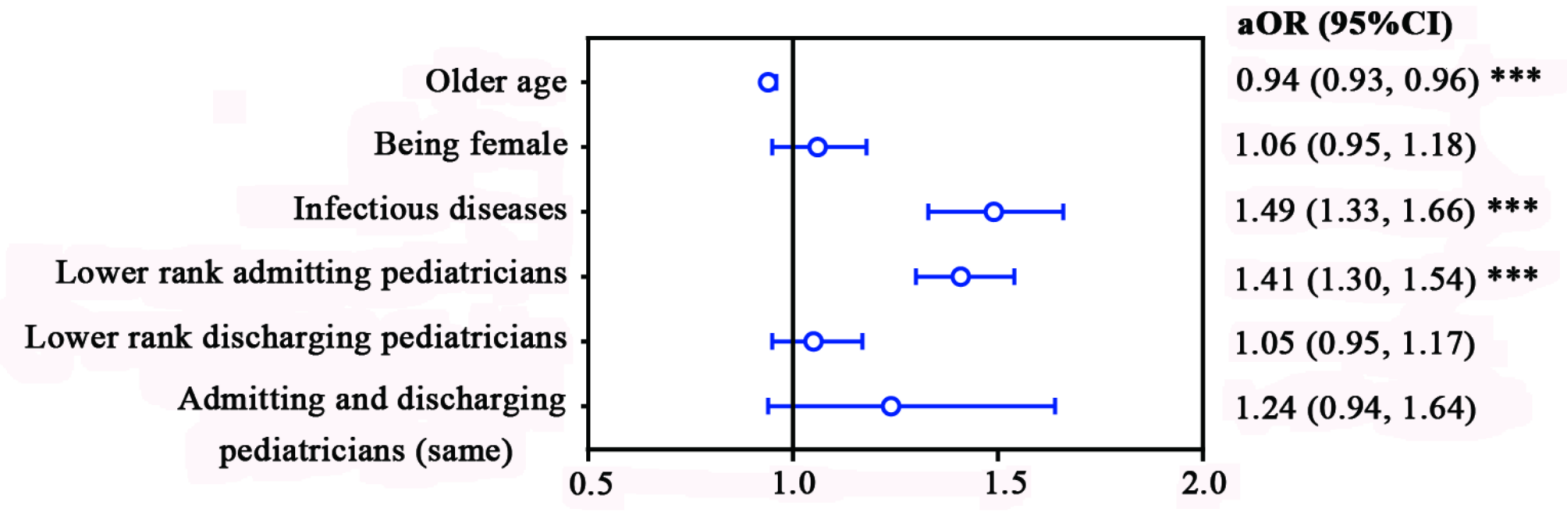
Multiple (multivariable) logistic regression analysis of factors associated with diagnosis discordance. Variables used in the regression for comparison included the older age group vs. younger age group; female vs. male; infectious diseases vs. others; lower-rank vs. higher-rank admitting pediatricians; lower-rank vs. higher-rank discharging pediatricians; same vs. different pediatricians for admission and discharge. X-axis, log-odds scale

Multiple (multivariable) logistic regression analysis (Fig. 3) also predicted a higher risk of diagnostic discordance with infectious disease cases and lower rank admitting pediatricians (aOR, 95%CI: 1.49, 1.33–1.66 and 1.41, 1.30–1.54, respectively, *P*s < 0.001) and lower risk with older children (aOR, 95%CI: 0.94, 0.93–0.96, *P* < 0.001).

### Clinical management and outcomes (Table 2)

Most cases were admitted by attending pediatricians (66.6%, 3582/5381) and discharged by attending and resident pediatricians (55.2%, 2973/5381; and 42.7%, 2300/5381, respectively). Different pediatricians were responsible for the admission and discharge of the same patient in most cases (95.8%, 5156/5381).

Antibiotics were prescribed to 41.3% (2220/5381) of cases. Compared with diagnostic concordant cases, discordant cases had a higher antibiotic prescription rate (32.6%, 890/2732 vs. 50.2%, 1330/2649; OR2.09, 95%CI: 1.87–2.33; *P* < 0.001), longer duration of antibiotic use (*P* = 0.02), and longer length of stay in hospital (*P* < 0.001).

Most cases (71.1%, 3827/5381) were discharged with complete recovery. Although there was no significant difference in clinical outcomes, higher hospital expenses were observed with diagnostic discordance (*Ps* < 0.001).

## Discussion

In this study, we present the prevalence of diagnostic discordance, susceptible clinical conditions, factors responsible for discordance, and associated adverse outcomes in the pediatric practice in the Chinese context and potential problems with ICD-based diagnostic consistency assessment.

### Justification of diagnostic consistency assessment and classification approach

The ICD-10-based assessment method we used is simple and suitable for studying a large sample size. With the primary discharge diagnosis as the reference, we considered all admission diagnoses, including primary, provisional, and differential diagnoses, for comparison to understand the factors behind concordant as well as discordant diagnoses. This approach is different from the previous studies [16, 18], in which only primary admission diagnoses were used for comparison.

### Prevalence of diagnostic discordance and associated ICD-10 groups and clinical conditions

The rate of diagnostic discordance or “unmatch” (49.2%) in this study is considerably higher than the reported ranges (18 − 28% for all ages or 34% for pediatric cases) admitted via emergency departments in previous studies [11, 12, 18]. Since diagnostic problems are known to prevail in emergency admissions, the high discordance rate in our pediatric cases at inpatient admission warrants further investigation into possible diagnostic errors.

Infections, cardiovascular diseases, and cancers are the conditions known to be highly susceptible to diagnostic error for all ages [22]. But for children, common conditions resulting in insurance claims are meningitis, gastroenteritis, pneumonia, appendicitis, sepsis, and malignancy [23]. Likewise, in this study, three ICD-10 groups—the infectious diseases (A) and the diseases of the nervous system (G) and the respiratory system (J)—were most susceptible to diagnostic discordance (Fig. 1), with infectious diseases occupying top 10 discordant ICD-10 codes (Fig. 2).

### Factors associated with diagnostic consistency in pediatric practice

Diagnostic consistency rates reported in previous studies vary with the study population, clinical condition, and the characteristics of physicians or coders [8, 11, 12, 18]. In our pediatric setting, in addition to those factors, the coding process and ICD-based assessment appeared as confounders for both discrepant and concordant diagnoses as discussed hereafter.

#### Susceptible patients

Contrary to older age being a risk factor in the adult population [8, 12], younger age is significantly associated with discordant diagnosis in our pediatric population. Age-specific prevalence and manifestations of certain diseases, especially infections in the respiratory and nervous systems, could explain younger age as the risk factor in children.

#### Susceptible clinical conditions

The manifestations of certain diseases are non-specific initially, and thus a definitive diagnosis is not possible in ambulatory as well as urgent care settings. Therefore, at times patients might be admitted for observation or further diagnostic workup. We noted two clinical conditions that are susceptible to discordant diagnosis: diseases with general or vague initial presentations and diseases with pathognomonic signs, both, nevertheless, requiring confirmatory testing to reach a specific diagnosis. These two conditions were represented by infectious diseases, claiming the top 10 discordant clinical conditions in this study. Our regression model also predicted that pediatric infections are more likely to fall under the discordant category even after controlling for the patient`s age and sex and physician factors. Whereas the nature of the diagnostic process that involves the identification of the etiologic agent in most cases of infection may be the main reason for discordance, diagnostic errors could also be a contributor because misdiagnosing viral infections as bacterial illness is the most common diagnostic error in pediatric practice [2], which is exemplified by bacterial pneumonia topping discordant ICD-10 codes (Fig. 2). One reason is that there is currently no reliable test to differentiate bacterial from viral infection with high accuracy clinically. Although metagenomic next-generation sequencing (mNGS) shows a higher sensitivity for pathogen identification, its clinical value is relatively limited due to the high cost [24].

In a study with emergency admissions in Hong Kong, the rate of unmatched diagnoses was 17% for specific provisional diagnoses and up to 52% for non-specific provisional diagnoses [12]. The diagnostic discordance rate for non-specific provisional diagnoses (i.e., ICD-10 group R symptoms/signs, Fig. 1) in this study is much lower at 36.8%, with a discordance-to-concordance ratio of 0.4. It is reasonable that non-specific diagnoses are more common in emergency admissions.

#### Physician factors

In a multisite study with 726 American pediatricians, over half of them self-reported as having made a diagnostic error at least once or twice per month [10]. Attending and resident pediatricians being significantly associated with diagnostic discordance in this study is concerning because they were responsible for most admissions and discharges (Table 2). Work overload could be a plausible contributor because although China has 4 pediatricians per 10, 000 children as of 2014 [25], which is much higher than the average of Southeast Asian countries where the ratio is 4 pediatricians per 100,000 children [26], they are concentrated in big cities and tertiary hospitals. Thus, Chinese pediatricians in tertiary hospitals are overstretched [25]. In our tertiary hospital, one pediatrician is responsible for 50–100 outpatients and 10–12 inpatients per typical day.

#### Coding process

Despite discharge diagnosis being considered the gold standard description of a patient`s health problem, the primary discharge diagnoses are reportedly coded inaccurately in up to 55% [27]. Coding errors may arise from physicians` attestation errors (during clarification requested by the coder for final code assignment) or coder-level errors such as miscoding (assigning a generic code when information is available for a more specific code), misspecification (misalignment of primary diagnosis with the evidence in the record), resequencing (coding diagnoses reversely), upcoding (assigning codes of higher reimbursement value), or unbundling (coding for all the separate parts of a diagnosis instead of assigning a code for the overall diagnosis) [28]. There were cases of suspected miscoding, resequencing, upcoding, and misspecification leading to diagnostic discordance in this study with representative examples shown in Table 1.

#### Diagnostic consistency assessment approach

Three types of matching (complete, partial, or no match) between admission and discharge codes could arise from errors other than that of diagnosis as discussed with reference to some examples in Table 1 henceforth.

- *Complete match*: Lack of specific diagnosis in completely matched cases, such as case 1: bronchopneumonia (J18.0) or case 5: hyperpyrexia (R50.900), raises the possibility of an error in physician`s note, miscoding, failure to establish a specific diagnosis, or parental disagreement with costly diagnostic testing, which is not uncommon for financially constrained families in China.
- *Partial match*: Whilst most partially matched cases were the result of a typical diagnostic process (e.g., Case 6: Pneumonia, unspecified (J18.900) at admission and severe viral pneumonia (J18.802) at discharge), cases like Case 9: herpangina (B08.501) at admission and hand-foot-mouth disease (B08.401) at discharge, was a diagnostic error.
- *No match*: Almost all no matched cases were supposedly due to diagnostic or coding errors as described earlier, but no match between “sepsis (A41.9)” at admission and “Candidal sepsis (B37.7)” at the discharge of Case 10 should not specify any error.

Taken together, all these examples illustrate that even perfect matches or mismatches between discharge and admission diagnoses may not indicate the absence or presence of diagnostic errors. Given the complex nature of the diagnostic process, ascertainment of errors could be impossible [1] and thus we would not further attempt to discern diagnostic errors from diagnostic discordance.

### Outcomes associated with diagnostic discordance

In addition to the longer length of stays and higher medical expenses reported in previous studies [8, 14, 17, 19], higher rates and longer duration of antibiotic prescription due mostly to infections in the respiratory, gastrointestinal, and nervous systems were the adverse outcomes from diagnostic discordance in this study.

### Study limitations

One major limitation in this study is that given a legally sensitive nature, we could not verify the rate of intentional miscoding, and thus the coders` adherence to coding ethics, as well as the medicolegal consequences of diagnostic discordance. Also, since we could not accurately categorize and present the observed coder-level errors due to multiple possibilities in many cases (Table 1), we cannot provide specific recommendations for improvement.

In summary, investigating diagnostic consistency by using the ICD system is objective, less labor-intensive, and less error-prone, but the accuracy of consistency rests on coding accuracy and consistency classification criteria. Interpretation of discordance could be problematic in infectious diseases for which pathogen identification is required for management, leading to mismatched codes. Contingent upon the nature of the clinical condition, a discordance between admission and discharge ICD-10 codes may indicate the presence of a potential diagnostic error, coding error, code manipulation, or the normal/typical diagnostic process with or without significant impact on clinical outcome. Therefore, the ICD code should not be used as a stand-alone assessment tool for diagnostic consistency but rather as a guide for further investigation of high-risk clinical conditions, for instance, following the Safer Dx Instrument with a 13-item checklist to identify the diagnostic problems for quality improvement [29].

## Conclusions

This study denotes a considerably high rate of discordance between admission and discharge ICD-10 diagnostic codes with a higher and longer prescription of antibiotics, a longer length of stay, and higher medical expenses as the adverse outcomes among Chinese pediatric inpatient cases. Infectious diseases were identified as high-risk clinical conditions for discordance. Considering potential diagnostic and coding errors, departmental investigation of preventable diagnostic discordance is suggested for quality health care and preventing potential medicolegal consequences. Given that infectious diseases were subject to the highest diagnostic discordance, the application of mNGS may be considered for selected cases.

## Electronic supplementary material

Below is the link to the electronic supplementary material.

Supplementary Material 1

Supplementary Material 2

## Data Availability

All data generated or analyzed during this study are included in this published article and its supplementary information files.

## Abbreviations

EMR: Electronic Medical Records
ICD: International Classification of Diseases
mNGS: Metagenomic next-generation sequencing
