# Supplementary figures and images for: Diagnostic consistency between admission and discharge of pediatric cases in a tertiary teaching hospital in China
Source: BMC Pediatr. 2023 Apr 15;23:176. doi: 10.1186/s12887-023-03995-2 (PMC10105461; doi:10.1186/s12887-023-03995-2)

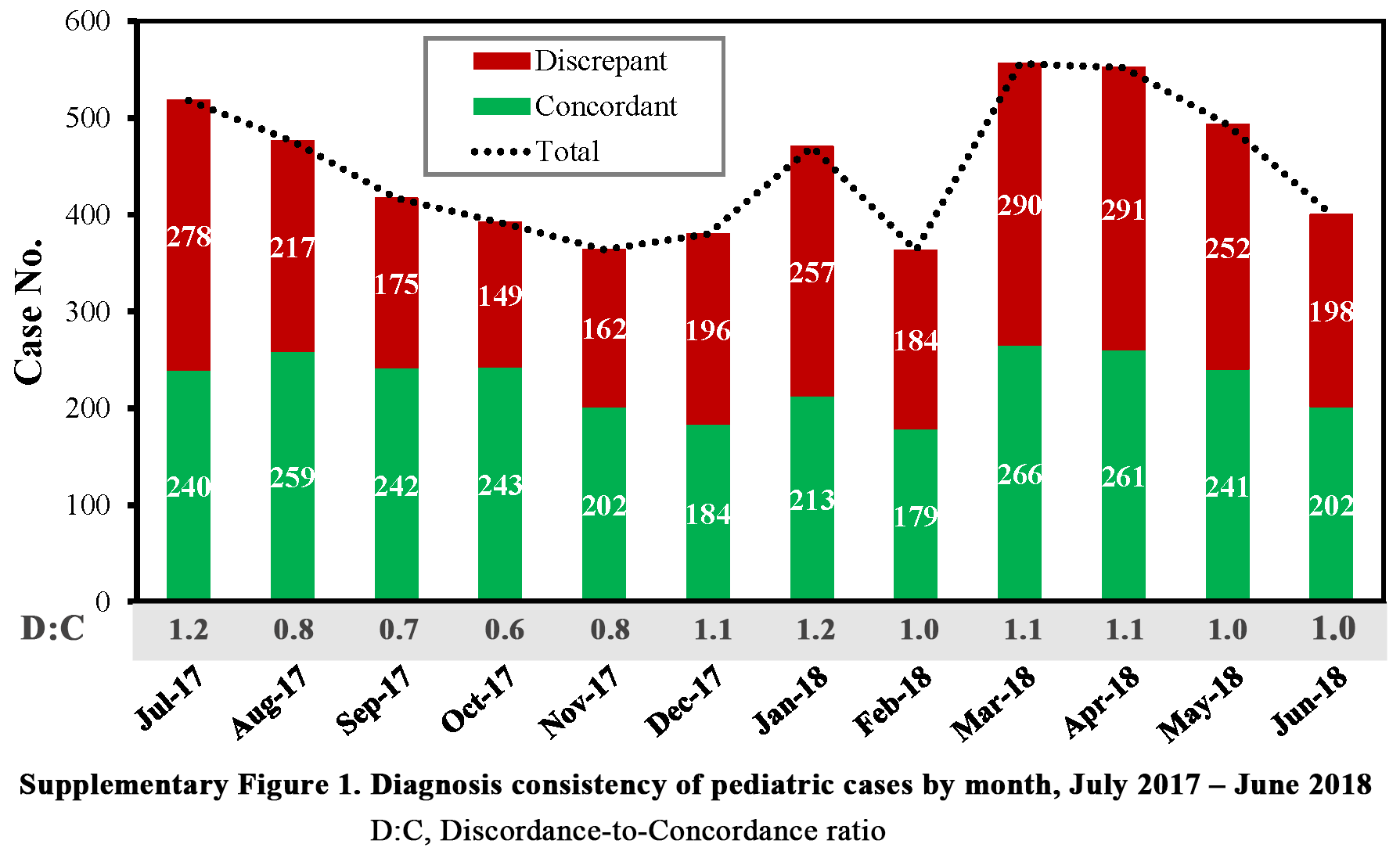

Supplement: Supplementary file 1 — Supplementary Material 1 [file 12887_2023_3995_MOESM1_ESM.tif]
