# Supplementary material for: Diagnostic consistency between admission and discharge of pediatric cases in a tertiary teaching hospital in China
Source: BMC Pediatr. 2023 Apr 15;23:176. doi: 10.1186/s12887-023-03995-2 (PMC10105461; doi:10.1186/s12887-023-03995-2)
Supplement: Supplementary file 2 — Supplementary Material 2 [file 12887_2023_3995_MOESM2_ESM.docx]

**Supplementary Table 1. Clinical data of pediatric inpatients and ICD-10-based diagnostic consistency**

| **Clinical data** | |  | **Matching Consistency^1^** | | |  | **Diagnostic Consistency** | | | | | | | | |
| --- | --- | --- | --- | --- | --- | --- | --- | --- | --- | --- | --- | --- | --- | --- | --- |
|  |  |  |  | | | *P*^4^ | **Flexible Criteria** | | *P*^4^ | OR (95%CI)^5^ | **Stringent Criteria** | | *P*^4^ | OR (95%CI)^5^ | |
|  |  |  |  |  |  |  | Concordant | Discordant |  |  | Concordant | Discordant |  |  |  |
|  |  | Total  (N = 5381) | No match^2^ n = 2649 (49.2%) | Partial match^3^ n = 145 (2.7%) | Complete match n = 2587 (48.1%) |  | Complete +  Partial match n = 2732 (50.8%) | No match  n = 2649 (49.2%) |  |  | Complete match n = 2587 (48.1%) | Partial +  No match n = 2794 (51.9%) |  |  |  |
| **Sex** | |  |  |  |  | 0.808 |  |  | 0.76 |  |  |  | 0.911 |  | |
|  | Male | 3207 (59.6) | 1573 (59.4) | 90 (62.1) | 1544 (59.7) |  | 1634 (59.8) | 1573 (59.4) |  | 1 | 1544 (59.7) | 1663 (59.5) |  | 1 | |
|  | Female | 2174 (40.4) | 1076 (40.6) | 55 (37.9) | 1043 (40.3) |  | 1098 (40.2) | 1076 (40.6) |  | 1.0 (0.9-1.1) | 1043 (40.3) | 1131 (40.5) |  | 1.0 (0.9-1.1) | |
| **Age range** (28 days - 15 years) | |  |  |  |  | **<0.001** |  |  | **<0.001** |  |  |  | **<0.001** |  | |
|  | ≥ 3 yr | 1853 (34.4) | 806 (30.4) | 57 (39.3) | 990 (38.3) |  | 1047 (38.3) | 806 (30.4) |  | 1 | 990 (38.3) | 863 (30.9) |  | 1 | |
|  | 2 ~ 3 yr | 686 (12.7) | 384 (14.5) | 24 (16.6) | 278 (10.7) |  | 302 (11.1) | 384 (14.5) |  | 1.7 (1.4-2.0) | 278 (10.7) | 408 (14.6) |  | 1.7 (1.4-2.0) | |
|  | 1 ~ < 2 yr | 1063 (19.8) | 584 (22.0) | 22 (15.2) | 457 (17.7) |  | 479 (17.5) | 584 (22.0) |  | 1.6 (1.4-1.8) | 457 (47.7) | 606 (21.7) |  | 1.5 (1.3-1.8) | |
|  | < 1 yr | 1779 (33.1) | 875 (33.0) | 42 (29.0) | 862 (33.3) |  | 904 (33.1) | 875 (33.0) |  | 1.3 (1.1-1.4) | 862 (33.3) | 917 (32.8) |  | 1.2 (1.1-1.4) | |
| **Admitting pediatrician** | |  |  |  |  | **<0.001** |  |  | **<0.001** |  |  |  | **<0.001** |  | |
|  | Chief | 285 (5.3) | 60 (2.3) | 10 (6.9) | 215 (8.3) |  | 225 (8.2) | 60 (2.3) |  | 1 | 215 (8.3) | 70 (2.5) |  | 1 | |
|  | Associate chief | 927 (17.2) | 396 (14.9) | 29 (20.0) | 502 (19.4) |  | 531 (19.4) | 396 (14.9) |  | 2.8 (2.1-3.8) | 502 (19.4) | 425 (15.2) |  | 2.6 (1.9-3.5) | |
|  | Attending | 3582 (66.6) | 1882 (71.0) | 90 (62.1) | 1610 (62.2) |  | 1700 (62.2) | 1882 (71.0) |  | 4.2 (3.1-5.6) | 1610 (62.2) | 1972 (70.6) |  | 3.8 (2.9-5.0) | |
|  | Resident | 587 (10.9) | 311 (11.7) | 16 (11.0) | 260 (10.1) |  | 276 (10.1) | 311 (11.7) |  | 4.2 (3.1-5.9) | 260 (10.1) | 327 (11.7) |  | 3.89 (2.8-5.3) | |
| **Discharging pediatrician** | |  |  |  |  | 0.810 |  |  | 0.518 |  |  |  | 0.462 |  | |
|  | Chief | 1 (<0.1) | 1 (<0.1) | 0 (0) | 0 (0) |  | 0 (0) | 1 (<0.1) |  | Not relevant | 0 (0) | 1 (<0.1) |  | Not relevant | |
|  | Associate chief | 107 (2.0) | 49 (1.8) | 3 (2.1) | 55 (2.1) |  | 58 (2.1) | 49 (1.8) |  | 1 | 55 (2.1) | 52 (1.9) |  | 1 | |
|  | Attending | 2973 (55.2) | 1450 (54.7) | 76 (52.4) | 1447 (55.9) |  | 1523 (55.7) | 1450 (54.7) |  | 1.1 (0.8-1.7) | 1447 (55.9) | 1526 (54.6) |  | 1.1 (0.8-1.6) | |
|  | Resident | 2300 (42.7) | 1149 (43.4) | 66 (45.5) | 1085 (41.9) |  | 1151 (42.1) | 1149 (43.4) |  | 1.2 (0.8-1.7) | 1085 (41.9) | 1215 (43.5) |  | 1.2 (0.8-1.8) | |
| **Admitting and discharging pediatricians** | | |  |  |  | 0.319 |  |  | 0.276 |  |  |  | 0.173 |  | |
|  | Same | 225 (4.2) | 119 (4.5) | 8 (5.5) | 98 (3.8) |  | 106 (3.9) | 119 (4.5) |  | 1 | 98 (3.8) | 127 (4.5) |  | 1 | |
|  | Different | 5156 (95.8) | 2530 (95.5) | 137 (94.5) | 2489 (96.2) |  | 2626 (96.1) | 2530 (95.5) |  | 0.9 (0.7-1.1) | 2489 (96.2) | 2667 (95.5) |  | 0.8 (0.6-1.1) | |
| **Clinical condition** | |  |  |  |  | **<0.001** |  |  | **<0.001** |  |  |  | **<0.001** |  | |
|  | Non-infectious diseases | 2938 (54.6) | 1291 (48.7) | 82 (56.6) | 1565 (60.5) |  | 1647 (60.3) | 1291 (48.7) |  | 1 | 1565 (60.5) | 1373 (49.1) |  | 1 | |
|  | Infectious diseases | 2443 (45.4) | 1358 (51.3) | 63 (43.4) | 1022 (39.5) |  | 1085 (39.7) | 1358 (51.3) |  | 1.6 (1.4-1.8) | 1022 (39.5) | 1421 (50.9) |  | 1.6 (1.4-1.8) | |
| **Antibiotic use (Yes)** | | 2220 (41.3) | 1330 (50.2) | 38 (26.2) | 852 (32.9) | **<0.001** | 890 (32.6) | 1330 (50.2) | **<0.001** | 2.1 (1.9-2.3) | 852 (32.9) | 1368 (49.0) | **<0.001** | 2.0 (1.8-2.2) | |
| **Duration of antibiotic use** (median day, IQR) | | 5 (4, 7) | 5 (4, 7) | 6 (4, 7) | 5 (4, 7) | **0.028** | 5 (4, 7) | 5 (4, 7) | **0.020** |  | 5 (4, 7) | 5 (4, 7) | **0.01** |  | |
| **Length of stay** (median day, IQR) | | 5 (3, 7) | 5 (4, 7) | 4 (3, 7) | 4 (3, 6) | **<0.001** | 4 (3, 6) | 5 (4, 7) | **<0.001** |  | 4 (3, 6) | 5 (4, 7) | **<0.001** |  | |
| **Outcome** | |  |  |  |  | 0.951 |  |  | 0.792 |  |  |  | 0.732 |  | |
|  | Recovered completely | 3827 (71.1) | 1880 (71.0) | 100 (69.0) | 1847 (71.4) |  | 1947 (71.3) | 1880 (71.0) |  | 1 | 1847 (71.4) | 1980 (70.9) |  | 1 | |
|  | Discharged with partial recovery | 1551 (28.8) | 768 (29.0) | 45 (31.0) | 738 (28.5) |  | 783 (28.7) | 768 (29.0) |  | 1.0 (0.9-1.1) | 738 (28.5) | 813 (29.1) |  | 1.0 (0.9-1.2) | |
|  | Transferred to another hospital | 1 (<0.1) | 0 (0) | 0 (0) | 1 (<0.1) |  | 1 (<0.1) | 0 (0) |  | Not relevant | 1 (<0.1) | 0 (0) |  | Not relevant | |
|  | Died in hospital | 2 (<0.1) | 1 (<0.1) | 0 (0) | 1 (<0.1) |  | 1 (<0.1) | 1 (<0.1) |  | 1.0 (0.1-16.6) | 1 (<0.1) | 1 (<0.1) |  | 0.9 (0.1-14.9) | |
| **Hospital fees** (median RMB, IQR) | | | | | | | | | | | | | | |  |
|  | Total | 3411 (2282, 5061) | 3915 (2652, 5784) | 3010 (1985, 4819) | 2958 (2015, 4282) | **<0.001** | 2965 (2011, 4290) | 3915 (2652, 5784) | **<0.001** |  | 2958 (2015, 4282) | 3871 (2591, 5736) | **<0.001** |  | |
|  | Drugs only | 509 (249, 888) | 636 (332, 1040) | 393 (178, 751) | 411 (204, 702) | **<0.001** | 411 (202, 706) | 636 (332, 1040) | **<0.001** |  | 411 (204, 702) | 617 (317, 1025) | **<0.001** |  | |

Categorical variables (sex, age group, clinical condition, type of pediatricians, antibiotic use, outcome) shown as n (%), analyzed by the Chi-square test; Continuous variables (duration of antibiotic use, length of hospital stay, and hospital fees) shown as median (IQR), analyzed by the Mann-Whitney U test. 1RMB = 0.15 US$; ^1^ Complete match (all first 4 digits of ICD-10 code matched), partial match (at least first 3 digits matched), no match (first 3 digits unmatched); ^2^ Including secondary and differential diagnoses; ^3^ including miscoding (assigning a generic code when information is available for a more specific code), misspecification (misalignment of primary diagnosis with the evidence in the record), resequencing (coding diagnoses reversely), upcoding (assigning codes of higher reimbursement value); ^4^ Concordant vs discordant; ^5^ Risk of discordance.
